# Interplay Between Air Travel, Genome Integrity, and COVID-19 Risk vis-a-vis Flight Crew

**DOI:** 10.3389/fpubh.2020.590412

**Published:** 2020-12-18

**Authors:** Sneh M. Toprani, Christopher Scheibler, Zachary D. Nagel

**Affiliations:** ^1^Department of Environmental Health, Harvard T.H. Chan School of Public Health, Boston, MA, United States; ^2^John B. Little Center for Radiation Sciences, Harvard T.H. Chan School of Public Health, Boston, MA, United States; ^3^Environmental and Occupational Medicine and Epidemiology Program, Harvard T.H. Chan School of Public Health, Boston, MA, United States

**Keywords:** flight attendant, pilot, COVID-19, coronavirus, DNA damage repair, air travel, aviation industry, genomic integrity

## Abstract

During air travel, flight crew (flight attendants, pilots) can be exposed to numerous flight-related environmental DNA damaging agents that may be at the root of an excess risk of cancer and other diseases. This already complex mix of exposures is now joined by SARS-CoV-2, the virus that causes COVID-19. The complex exposures experienced during air travel present a challenge to public health research, but also provide an opportunity to consider new strategies for understanding and countering their health effects. In this article, we focus on threats to genomic integrity that occur during air travel and discuss how these threats and our ability to respond to them may influence the risk of SARS-CoV-2 infection and the development of range of severity of the symptoms. We also discuss how the virus itself may lead to compromised genome integrity. We argue that dauntingly complex public health problems, such as the challenge of protecting flight crews from COVID-19, must be met with interdisciplinary research teams that include epidemiologists, engineers, and mechanistic biologists.

**Graphical Abstract:**
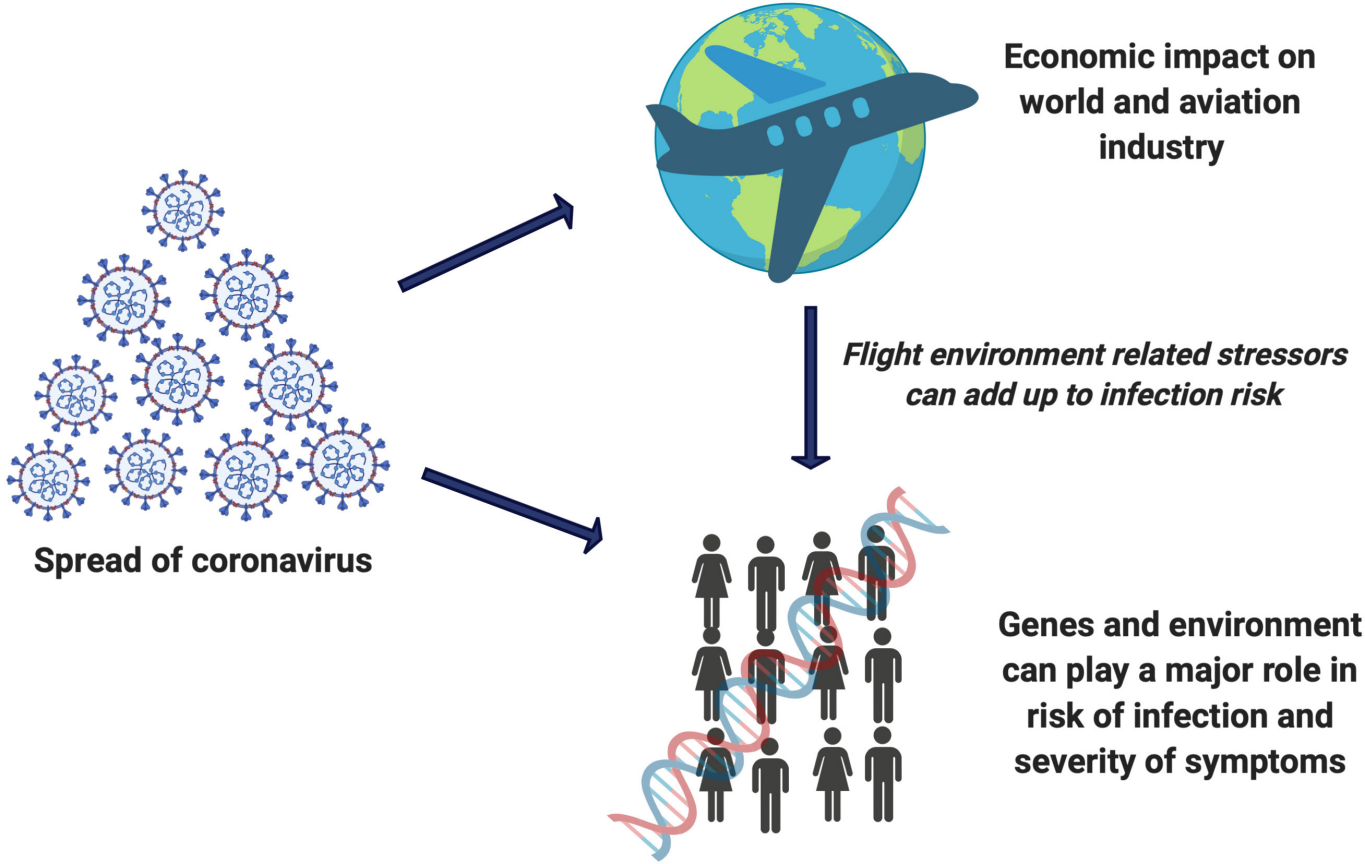
SARS-CoV-2 risk is an add-on entity to the list of flight related damaging effects which can impact the genome integrity of flight crew.

## Introduction

Understanding and solving public health crises require consideration of diverse inputs spanning from the molecular to the global level. The current COVID-19 pandemic illustrates this dynamic particularly well in the context of air travel, which entails exposure to a complex variety of potentially hazardous agents, some of which are likely responsible for the excess risk for cancer and other diseases in flight crews (1–8).

Ensuring safety during air travel represents a large and complex challenge. Approximately 4.1 billion passengers travel by air each year (9). There are estimated to be over 140,000 airline pilots employed internationally, and approximately 120,000 flight attendants are employed just in the U.S. (10). On 11th March 2020, the World Health Organization declared COVID-19, the disease caused by severe acute respiratory syndrome coronavirus 2 (SARS-CoV-2) to be a pandemic. The disease has now touched nearly every aspect of our lives and has severely impacted the aviation industry (11). Unprecedented flight cancellations, restriction of non-essential travel, border closures, and policies taken in response to the pandemic have hit the travel industry very hard (12). With the uncertainty about the development and eventual efficacy of a vaccine for COVID-19, social distancing and engineering solutions such as the usage of personal protective equipment (PPE) are pivotal measures to lower the risk of COVID-19 transmission. New guidelines have been implemented by the Center for Disease Control and Prevention (CDC) and Federal Aviation Administration (FAA) for air travel during the COVID-19 pandemic, but airlines still struggle to balance scheduling of fewer flights with maximizing social distancing (13, 14). Further complicating matters, air travel demand is in flux, and social distancing in airplanes may be impossible in some aircraft due to seat configuration. Other necessary aspects of air travel that occur outside the airplane, such as passing through Transport Security Administration (TSA) precheck, can also potentially increase the risk of COVID-19 exposure for flight crew. Thus, every single aspect during air travel needs to be considered in order to avoid increased risk of exposure and spread of SARS-CoV-2.

The high frequency of asymptomatic SARS-CoV-2 carriers and the period between exposure and infection of up to 2 weeks pose an acute challenge for estimating the risk of transmission in general, and during the air travel in particular. Furthermore, we do not yet understand why the virus has such disparate impacts on different people. Although some clues are beginning to emerge and are discussed in more detail below, relatively little is known about the role of environmental exposures and inter-individual biological variation with regard to susceptibility to viral infection or development of severe symptoms. We note, however, that several of the exposures that are associated with greater risk of severe disease include agents that compromise genome integrity (15, 16). Genome integrity refers to the protection of DNA from damage and mutations that can impact cellular function or lead to cell death (17). Compromised genome integrity is linked to various health risks (17, 18). This article briefly highlights the possible role of genome integrity in the etiology of COVID-19 and other diseases that are of concern for flight crew. We recommend a multidisciplinary paradigm for advancing our understanding of complex public health problems by focusing on key biological pathways, in this case genome integrity.

## Air Travel and Genome Integrity

Flight crew are subjected to diverse sources of flight related exposures that may pose health risks. These exposures include physical stressors (air pressure changes, vibration, changes in oxygen levels, prolonged immobility, aircraft noise); in-flight chemical exposures (pesticides, flame retardants); radiation (cosmic ionizing radiation, ultraviolet light) and biological changes (circadian rhythm disruption, mental and physical stress) (7, 19, 20). In addition, flight attendants are at higher risk of viral exposure due to their interactions with passengers and are expected to experience an increased work-related stress during the pandemic (21–23).

A growing body of literature suggests that flight-related exposures can directly or indirectly affect the human body. In particular, some of these exposures damage DNA, the genetic blueprint of the cell (24–26). DNA damage can lead to cell death or mutations, which are key biological mechanisms underlying a variety of diseases. To avoid cell death and mutagenesis, DNA repair mechanisms in human cells remove DNA damage and thereby maintain genome integrity (27–30). Inefficient DNA repair in humans has also been associated with susceptibility to several diseases including cancer and immune disorders that predispose to infection (8, 31–34). Epidemiological studies have documented excess cancer risk in flight attendants and pilots (1–8), indicating that compromised genome integrity may be a particular problem for this population. Although not all of the agents to which flight crew are exposed are known DNA damaging agents, some flight crew-based research studies have demonstrated that exposure to cosmic ionizing radiation, which damages DNA, is associated with elevated cancer risk (35–40). Additional evidence comes from studies that have found links between cosmic ionizing radiation exposure and reproductive health risk (2, 41, 42), but the underlying biological mechanism has not yet been elucidated.

One testable hypothesis is that DNA damage and alterations in repair responses due to flight related exposures are responsible for the diminished genomic integrity that leads to increased risk of cancer and perhaps other diseases among flight crew. Notably, viral infections also pose a threat to genome integrity (43) and there is evidence suggesting that the severity of disease caused by respiratory RNA viruses may be related to the extent of DNA damage induced during and after infection (44). Since the ability to repair DNA damage would influence the extent of DNA damage that accumulates during infection, and since DNA repair pathways play a central role in immune function, it is possible that DNA repair capacity impacts susceptibility to severe COVID-19. Thus, some of the same principles that are increasingly well-understood in the context of genome integrity and cancer risk may also apply to understanding the origins of inter-individual differences in susceptibility to viral infections and the development of severe symptoms, but this idea has yet to be explored.

## COVID-19 Risk and Genome Integrity

SARS-CoV-2 is an RNA virus that hijacks host cellular mechanisms during its lifecycle (45, 46). RNA viruses can trigger a multitude of cellular changes including activation of the DNA damage response, DNA replication stress, dysregulation of the cell cycle, induction of apoptosis, and several other stress responses (47–49). In the case of SARS-CoV-2, alterations in cellular processes that promote viral replication also lead to activation of oncogenic pathways (50), which raises questions regarding the potential for SARS-CoV-2 to promote cancer (51). As is the case for cancer risk (29, 34, 51) genetic background contributes toward inter-individual variation in susceptibility and treatment outcomes for respiratory disease (52). In the case of SARS-CoV-2, polymorphisms in the gene encoding angiotensin converting enzyme 2 (ACE2) have been associated with variation in susceptibility to SARS-CoV-2 infection (53, 54). The etiology of SARS-CoV-2 infection and severe COVID-19 is multifactorial and can be attributed to polymorphisms immune system genes (55, 56), viral mutations (57), variation in the respiratory microbiome (58). Although DNA repair has not been studied in the context of SARS-CoV-2 susceptibility, limited studies that have been conducted in other infectious disease contexts suggest that polymorphisms in DNA repair genes may affect host responses to pathogens (59, 60).

The potential interaction between cancer and COVID-19 outcomes remains largely unexplored and may provide additional insights into a possible shared role for genome integrity in the etiology of both diseases. A study among 1,590 COVID-19 patients in China revealed cancer to be an important risk factor for health endpoints associated with severe COVID-19 (61), raising important ethical questions regarding the prioritization of treatment (62). In further support of a link between cancer and COVID-19, an analysis by WHO found the fatality rate among COVID-19 infected patients with cancer was double the rate for COVID-19 patients without cancer (51). This excess mortality may be attributed at least in part to compromised immune function in cancer patients due to treatments and the effects of the disease itself. However, the biological mechanisms underlying these associations remain unclear. Given the well-established relationship between cancer risk and inter-individual variation in DNA repair capacity (34) and given the potential role for persistent DNA damage in the pathogenesis of COVID-19, an intriguing possibility is that DNA repair capacity might play a role in the severity of COVID-19 infection and treatment outcomes. A more complete understanding of how SARS-CoV-2 impacts genome integrity would provide valuable information for developing potential DNA damage and repair biomarkers that could help to assess an individual's risk infection and severe illness due to SARS-CoV-2 and other viral infections that may be influenced by genome integrity pathways.

## Current and Future Approaches to Risk Mitigation

Despite aggressive efforts to minimize SARS-CoV-2 risk during air travel, this exposure joins a long list of others that cannot be entirely eliminated for those who must travel. In-flight transmission risk is still not completely understood, and several studies have shown compelling evidence of validated in-flight COVID-19 mass transmission from past in-flight events (63, 64). The advanced particle filtration and air exchange systems used in commercial aircraft should decrease transmission risk, which was supported by a recent in-flight aerosol dispersion study that found minimal aerosol exposure risk even during long duration flights (65). In-flight COVID-19 risk is likely impacted by differences between aircraft type, ventilation systems and real-world flight environments as compared to experimental flight conditions, however one factor that appears to have a noteworthy impact on in-flight transmission is use of face-masking during air travel (66). For the time being, global, one-size-fits-all preventive measures are the mainstay of efforts to protect the public. Hand washing, masks, and other PPE (13), together with temperature checks, touchless check-in, one-way boarding (back to front), plexiglass shields and empty middle seats in aircraft, are among the available strategies for mitigating air travel-related spread of SARS-CoV-2 (67). Additional measures can likely be developed to augment these strategies. Improved screening tools can be developed to upgrade the existing non-laboratory-based methods for detecting COVID-19 infected individuals and ensuring asymptomatic infectious individuals do not travel. Coordination within airports is also essential for reducing the spread of SARS-CoV-2 infection among airport employees such as ticketing staff, TSA, security personnel, airport workers and others. In the US, TSA security procedures have already been updated and implemented to minimize the risk of COVID-19 transmission (68).

Looking forward, the myriad exposures encountered by flight crews will remain for the foreseeable future, necessitating new approaches for understanding and mitigating their potential health effects. Even after the COVID-19 pandemic passes, lessons learned during this time should inform preparedness for similar events in the future. Some strategic measures available to individuals that have been proposed include avoiding itineraries that may disrupt circadian rhythm (69) or using antioxidants to counteract the production of free radicals produced as a consequence of viral infection or exposures encountered during travel (70). Many historical areas of concern for adverse flight environment exposures such as ultraviolet (UV) radiation have been mostly mitigated through upgrades in aircraft materials or systems (71). However, a comprehensive understanding of the biological mechanisms underlying the excess risk of disease in flight crews will be needed in order to develop truly personalized prevention strategies that take into account individual exposures and inter-individual differences in responses to exposures.

There is a need for multidisciplinary approaches to address this issue and gather information about the impact of air travel on genomic integrity. Although many factors contribute to disease risk, one promising approach toward improved and more personalized prevention is to focus on genome integrity, a fundamental biological pathway that links many of the exposures encountered by flight crew. Well-designed studies should make use of dosimetry or sensors to quantify as many occupational exposures as possible. Comprehensive exposure analysis will enable efforts to determine the effect of complex mixtures. By joining these analyses with laboratory based mechanistic studies using genomics approaches and functional assays in biospecimens and model systems, we can improve our understanding of how DNA damage and repair influence health outcomes in flight crew. Such studies will provide a foundation for developing personalized prevention or risk assessment profiles for the flight crew that may also benefit passengers.

## Conclusions

The COVID-19 pandemic has severely impacted the global economy, affecting several industries and having a ripple effect on every aspect of human life. The complexity and magnitude of the challenge of responding to COVID-19 is exemplified by the multiple, in some cases ill-defined risks associated with air travel. Because these risks are unavoidable for some, it appears the only way out is through. A multidisciplinary approach toward COVID-19 mitigation to support the safe and secure return of normal air travel operations is already underway (72). There still exist many unraveling puzzles regarding the biological basis for infection risk in humans that need to be explored and could strongly influence the preventive measures implemented by airlines in the future. Activation of host DNA damage responses are associated with RNA virus replication (46, 47), raising the possibility that inter-individual differences in DNA repair capacity may play a role in susceptibility to severe COVID-19 symptoms. DNA damage and repair biomarkers may provide insights into an individual's risk toward viral infection or poor outcomes. Better understanding of the etiology of COVID-19 will shed light on optimal approaches for limiting COVID-19 spread and may inform preparedness for future pandemics.

Research aimed at understanding individual disease susceptibility following flight-related exposures can assist policy makers in making data-driven decisions regarding travel regulations. Efforts to understand the biological mechanisms underlying disease as they operate in a real world context must be a central element of this research. This form of mechanistic epidemiology is sometimes perceived to be at odds with the conventional reductionist approach to biology, which favors the isolation of variables and experimentation with the simplest possible model. As a result, there will be a need for funding agencies to promote work that follows this paradigm. To achieve success, there is an utmost need to establish collaborative working platforms among researchers, engineers, clinicians and the pharmaceutical industry to identify new strategies for reducing the risk of COVID-19 during air travel. In turn this will help the aviation industry to develop measures for safeguarding passengers and aircrew during air travel. Although we have chosen in this article to focus on the complex interplay of COVID-19 and threats to genome integrity during air travel, the interdisciplinary mechanistic epidemiology approach we favor is applicable to studying COVID-19 in other settings, and for unraveling complex public health problems in general.

## Data Availability Statement

The original contributions generated for this study are included in the article/supplementary materials, further inquiries can be directed to the corresponding author/s.

## Author Contributions

ST: expert on biological DNA damage and repair mechanisms. CS: expert on flight-related exposures and overall medical/clinical effects and outcomes. ZN: expert on DNA damage and repair and drafting and editing. All authors contributed to the article and approved the submitted version.

## Conflict of Interest

The authors declare that the research was conducted in the absence of any commercial or financial relationships that could be construed as a potential conflict of interest.
